# The impact of dietary supplementation with tannic acid on the digestion of nutrients, rumen microflora, rumen fermentation, and methane production in Liuyang black goats

**DOI:** 10.5713/ab.25.0114

**Published:** 2025-09-30

**Authors:** Ying Yun, Ting Liu, Huihui Liu, Hui Zhang, Faming Pan, Lijing An, Xinji Wang, Guoyan Xu, Qiangwen Gu, Chen Zheng

**Affiliations:** 1 College of Animal Science and Technology, Gansu Agricultural University, Lanzhou, China; 2 Gansu Provincial Health Commission, Lanzhou, China; 3 Institute of Animal & Pasture Science and Green Agriculture of Gansu Academy of Agricultural Science, Lanzhou, China; 4 Animal Husbandry and Veterinary Station of Nawu Town, Hezuo, China; 5 Animal Husbandry and Veterinary Workstation of Minqin County, Minqin, China; 6 Animal Husbandry and Veterinary Workstation of Datan Town, Minqin County Agriculture and Rural Bureau, Minqin, China; 7 Animal Husbandry and Veterinary Workstation of Heli Town, Gaotai, China

**Keywords:** Methane, Nutrient Digestion, Rumen Fermentation, Rumen Microorganism, Tannic Acid

## Abstract

**Objective:**

This study investigated the effects of dietary tannic acid on methane production, nutrient digestibility, rumen fermentation, and rumen microbiota in Liuyang black goats.

**Methods:**

Twelve adult goats were randomly assigned to two groups: a control group and a treatment group that received 2% tannic acid in their diet. The experiment consisted of two stages, each comprising a 10-day adaptation period followed by and a 5-day sampling phase. Methane emission was measured using a mobile open-circuit respirometry system, while rumen fluid samples were analyzed for volatile fatty acids, ammonia nitrogen, and microbial composition by 16S rRNA sequencing.

**Results:**

The results indicated that the tannic acid significantly reduced overall methane emission (p<0.05), methane per dry matter intake (p<0.05), acid detergent fiber intake (p<0.05), and neutral detergent fiber intake (p<0.05). Microbial analysis showed increased relative abundance of *Firmicutes* (p<0.05) and decreased *Methanobrevibacter* and *Prevotella*. Before feeding, tannic acid led to a significant increase in the concentration of valerate in the rumen fluid (p<0.05), while the acetate to propionate ratio was significantly decreased (p<0.05). Three hours post feeding, the concentrations of both butyrate and valerate were significantly increased (p<0.05).

**Conclusion:**

Dietary tannic acid effectively reduced methane emission and enhanced feed efficiency in Liuyang black goats by modifying rumen fermentation and microbial activity. These findings indicate the potential of tannic acid as a sustainable feed supplement for ruminants; nevertheless, the long-term effects on health and production necessitate more research.

## INTRODUCTION

Mitigating methane emission, a potent greenhouse gas, is crucial for limiting global warming. A significant portion of anthropogenic methane is produced by livestock, particularly from ruminants, due to enteric fermentation. Given that methane has a long atmospheric lifetime of approximately 12 years, it is an ideal target for near-term climate action, as emphasized by the Global Methane Pledge initiative. To effectively reduce methane emission from livestock, strategies must be both scientifically sound and feasible (https://www.globalmethanepledge.org). Enteric fermentation is a major contributor to atmospheric methane, generating approximately 87 to 90 teragrams annually. This methane not only contributes to global warming but also results in a significant loss of 2% to 12% of dietary energy [1]. Enteric methane production is influenced by the rumen microbiome, diet, and animal interactions. Therefore, minimizing these emissions while improving feed conversion efficiency and nutrient utilization is essential for sustainable livestock production. Various mitigation strategies, including genetic selection, dietary feed additives, and chemical supplements, have been explored to reduce methanogenesis. By addressing enteric methane emission, we can both mitigate climate change and enhance dietary energy efficiency in livestock systems [2].

Numerous studies have highlighted the potential of plant secondary compounds, such as tannin, saponins, and essential oils, as alternative feed additives to modify ruminal fermentation, enhance antimicrobial activity, influence feeding behavior, improve animal productivity, and mitigate methane production [3]. Among these, tannin has emerged as a promising option for enteric methane mitigation [4]. However, the impact on animal production efficiency also requires further evaluation. It is essential to assess the effects of reducing methane emission in terms of dry matter intake (DMI), microbial activity, and rumen fermentation efficiency, including the acetate-to-propionate ratio [5]. Tannic acid, a polyphenolic metabolite, can modulate rumen fermentation and lower methane emission by binding to feed proteins, thereby protecting them from microbial degradation and increasing the availability of dietary protein and amino acids in the small intestine [6]. Additionally, the reduction in methane emission can occur through decreased fiber digestion and interference with rumen microorganisms [5]. Recent trials have demonstrated that moderate supplementation with condensed tannic acid extracts effectively decreases methane emission from rumen fermentation [7].

Previous research has demonstrated that tannic acid can influence rumen microbes, but comprehensive analyses using full-length V3–V4 16S rRNA sequencing are needed to fully understand its specific effects on rumen microbiota [2]. The rumen microflora is crucial for the performance and well-being of ruminants, yet there is currently a lack of extensive and conclusive data regarding the optimal levels of tannic acid supplementation for Liuyang black goats. Specifically, there is limited information on how tannic acid affects methane emission, nutrient digestibility, and the composition of the rumen microbiota in these animals. Therefore, this study aims to examine the effects of incorporating tannic acid into goat pellet feed on methane production, nutrient digestibility, and the rumen microflora of Liuyang black goats. The findings of this research will provide a theoretical basis for the development of environmentally friendly feed additives, supporting the dual objectives of achieving a low-carbon transformation and enhancing quality and efficiency in the livestock industry.

## MATERIALS AND METHODS

### Animal, diets and treatments

We used a controlled experimental design involving 12 male Liuyang black goats (body weight [BW] = 28±0.2 kg), sourced from the Institute of Subtropical Agriculture, Chinese Academy of Sciences in Changsha, P.R. China. The goats were divided into two treatment groups: (1) a control group with no supplemental tannic acid (CON), and (2) a treatment group receiving 2% tannic acid (TAN, g/kg diet on an as-fed basis), with 6 goats in each group. Each goat served as a replicate. The experiment was conducted in two stages, each lasting 20 days, with 6 goats in each stage (3 goats per treatment group). The tannic acid used was a commercially available compound obtained from *Rhus chinensis* Mill (purity 99.9%, Sinopharm Chemical Reagent Company). Each test lasted for 20 days, preceded by a 10-day pre-test period. On day 15 of each trial stage, we conducted respiratory metabolism tests using a single-cage respiratory chamber to measure the dynamic emissions of methane gas over a consecutive two-day period. All goats were housed in well-ventilated pens measuring 2.5 m×1.5 m and were fed twice daily at 08:00 and 17:00, following the feeding management protocols of the goat farm. They had *ad libitum* access to water throughout the experiment.

The experimental diets were developed and calculated following the Agricultural Industry Standard of the People’s Republic of China for raising meat goats (NY/T816-2021), which established the nutritional requirements for male goats weighing 30±0.2 kg on average. The experimental diet was a fully mixed pellet material, about 3.5 mm in diameter and 1–2 cm in length, provided by Gansu Runmu Biological Engineering. The composition and chemical composition of the experimental diets are shown in Table 1.

### Sample collection

The experimental design consisted of a 10-day adaptation phase followed by a 5-day sampling phase (days 10–15). During the sampling phase, daily measurements were conducted to gather essential data: (1) the quantity of feed provision and residues was recorded daily to determine DMI; (2) fecal output was collected using posterior plastic buckets; All collected samples underwent standardized processing procedures. Fecal samples were homogenized, and 10% (w/w) aliquots were retained for subsequent analysis. This integrated methodology facilitated a comprehensive assessment of nutrient utilization efficiency through mass balance analysis, allowing for a correlation between feed intake and excretion parameters. Additionally, BW was recorded every morning before feeding throughout the formal trial period, and average daily gain (ADG) was calculated based on the difference between initial and final BW. This comprehensive approach offers a robust framework for assessing both digestive performance and growth indicators in goats.

Rumen fluid samples were collected from the experimental sheep on day 13 of the trial, with sampling conducted over two consecutive days at two time points each day (before feeding and 3 hours after feeding). The rumen fluid was collected using a digestive tract cannula and immediately filtered through four layers of sterile cheesecloth. The pH of the filtered rumen fluid was measured promptly using a pH meter (pH-HJ90; Aerospace Computer Company). Subsequently, the filtered rumen fluid was aliquoted into cryovials of 2 mL, 5 mL, and 10 mL volumes and stored at −80°C for subsequent analyses of rumen microbiota, volatile fatty acids (VFAs), NH_3_-N concentration.

On day 15 of each phase, methane production was measured utilizing a mobile open-circuit respirometry system, which was adapted from the method developed by Wang et al [6]. For two consecutive days, respiratory metabolic methane was assessed in a closed breathing chamber. A gas flowmeter was installed within the chamber, maintaining an average airflow rate of 40 m^3^/h. Methane concentrations in the outlet gas and ambient air of each chamber were measured using an ultra-lightweight greenhouse gas analyzer (MIU-374-8; Los Gatos Research). The measurement cycle for methane concentration in each respiratory chamber was set at 60 minutes, which included a detection time of 9 minutes and a control setting of 9 minutes. Throughout the 24-hour respiratory metabolism assessment, a gas flow meter (C100L-CRWE-DD; SIYA) recorded the rate of methane production in LiuYang black goats. The measured methane concentrations were transmitted to a computer via sensors, and each chamber was calibrated with pure methane at a flow rate of 100 mL/min. Subsequently, the computer processed the data using the calibration parameters to determine the total amount and rate of methane production from the respiratory metabolism of LiuYang black goats. The methane emission data were averaged over the two consecutive days of measurement.

### Chemical analysis

The faeces were dried in an oven at 60°C for 48 hours and then ground to pass through a 0.45-mm screen using a stand model 4 Wiley Mill from Arthur H. Thomas in Philadelphia, PA, USA. The chemical composition was measured by determining the concentrations of dry matter (DM; method 930.15; AOAC [8]) and crude protein (CP; method 984.13; AOAC [8]), calcium and phosphorus (method 978.02, 946.06; AOAC [8]). The acid detergent fibre (ADF) and neutral detergent fiber (NDF) were analysed using an Ankom A200i Fiber Analyzer (ANKOM Technology) according to the methods of Van Soest et al [7]. The digestible energy (DE) was calculated using the method of the National Research Council (NRC) and tabular values for goats [9].

The VFAs of rumen liquid were quantified using high-performance gas chromatography (HPGC; GC-2014; Shimadzu) equipped with a hydrogen flame detector and a capillary column (Agilent Technologies; 30 m long, 0.32 mm diameter, 0.50 μm film). The temperatures for FID and column were 200°C and 120°C, respectively, with a carrier gas of nitrogen (purity 99.99%) with a flowing rate of 30 mL/min. The flowing rates of hydrogen and air were 30 mL/min and 300 mL/min, respectively. Before determination, 1 mL rumen fluid was mixed with 0.25 mL metaphosphoric acid (25%, w/v) and centrifuged at 15,000×g for 15 min at 4°C and the supernatant was used for VFAs determination. The ammonia concentration of the rumen fluid was determined using the phenol-hypochlorite colorimetric method, as described by Broderick and Kang, on the UV/vis spectrophotometer (UV-1801) made by Bei fen Rui li Analytical Instrument [10].

### DNA extraction, polymerase chain reaction amplification, and MiSeq sequencing

For microbial DNA extraction, rumen contents were collected from six black goats in each group. Total DNA was extracted from rumen contents using a QIAamp DNA Stool Mini Kit (QIAGEN), according to the manufacturer’s instructions. To assess the rumen microbial profiles, the V3-V4 region of the bacterial *16S rRNA* gene was amplified using the universal primers 515 F (5’-GTGCCAGCMGCCGCGGTAA-3′) and reverse primer 806 R (5′-GGACTACHVGGGTWTCTTA AT-3′) with barcode sequences [11]. Polymerase chain reaction (PCR) reactions were conducted with 15 μL of Phusion High-Fidelity PCR Master Mix (New England Biolabs), 0.2 μM of forward and reverse primers, and 10 ng of template DNA. Thermal cycling included initial denaturation at 98°C for 1 min, followed by 30 cycles of denaturation at 98°C for 10 s, annealing at 50°C for 30 s, and elongation at 72°C for 30 s and 72°C for 5 min [12]. Agarose gel electrophoresis (2%) was used to verify the amplicon size of each sample, and the PCR products were purified using a Qiagen Gel Extraction Kit (Qiagen). Sequencing libraries were generated using TruSeq DNA PCR-Free Sample Preparation Kit (Illumina) following the manufacturer’s recommendations, and index codes were added. Sample Preparation Kit (Illumina) following the manufacturer’s recommendations, and index codes were added. Library quality was assessed on the Qubit 2.0 Fluorometer (Thermo Fisher Scientific) and an Agilent Bioanalyzer 2100 system (Agilent Technologies). Finally, the library was sequenced on an Illumina NovaSeq platform (Realbio Technology Genomics Institute) using 250 bp paired-end reads.

### Bioinformatic analysis

Raw tags were obtained by splicing each sample read using FLASH (ver. 1.2.7, http://ccb.jhu.edu/software/FLASH/) [13]. QIIME (ver. 1.9.1, http://qiime.org/scripts/split_libraries_fastq.html) was used for strict quality control and filtering of raw tags to obtain clean tags [14]. The clean tags were compared with the species annotation database (https://github.com/torognes/vsearch/) to identify chimeric sequences that were removed and obtain the final effective tags [15]. Effective tags were clustered using the UPARSE software (ver. 7.0.1001, http://www.drive5.com/uparse/), and sequences with ≥97% similarity were assigned to the same operational taxonomic unit [16].

All the reads were identified and classified using the UCHIME algorithm and compared against the reference database [17]. The inter-group differences in the alpha and beta diversity indices were analyzed using R software. Chao1, Shannon, Simpson, ACE, and UniFrac distances were calculated using QIIME software (ver. 1.9.1) [18]. R software (ver. 4.3.0) was used to make box plots and principal coordinate analysis (PCoA) plots. PCoA was performed using the WGCNA, stats, and ggplot2 packages of R software was used to visualize microbiota association patterns [19]. Taxonomical annotation was performed using Mothur v 1.41.1 and by referring to Silva.nr.132 [20]. To identify microbial biomarkers associated with rumen microbiota composition, we performed linear discriminant analysis (LDA) effect size (LEfSe) through the Microbiome Process package in RStudio. This analysis systematically evaluated bacterial taxa across all taxonomic hierarchies from phylum to species level, with a stringent log LDA score threshold of ≥2.0 for biomarker significance. Subsequently, Spearman’s rank correlation analysis was conducted to quantify associations between rumen fermentation characteristics and two microbial parameters: (1) the relative abundance of the top 10 predominant bacterial genera, and (2) the phylum-level microbial composition profile.

### Calculations and statistical analysis

The apparent nutrient digestibility and ADG were calculated as follows:


(1) $$\begin{array}{l} \text{Apparent nutrient digestibility}=(\text{Nutrient intake}-\\ \text{Faecal nutrient output})/(\text{Nutrient intake})\times 100. \end{array}$$

(2) ADG (g/day)=(Final BW-Initial BW)/(Number of days measured)

Statistical analyses of the growth performance and apparent nutrient digestibility were performed using SPSS (IBM released 2019 and IBM SPSS Statistics for Windows, ver. 26.0; IBM) with the following linear model:


(3) $$Y_{\text{i}}=\mu +T_{i}+{ɛ}_{i}$$

Where *Y_i_* denotes the treatment mean over the 5-day collection period for the ith dietary group; *μ* is the overall mean; *T_i_* is the fixed effect of the two treatments (CON and TAN; *i* = 1 and 2); *ɛ_i_* is the random residual error.

The relative abundance of microbial bacteria and VFAs concentrations were compared using an independent-samples t-test in IBM SPSS Statistics for Windows, ver. 26.0 (IBM). The following model was:


(4) $$\text{t}=\frac{\bar{x_{1}}-\bar{x_{2}}}{S_{\bar{x_{1}}}-\bar{x_{2}}}$$

Where $\bar{x_{1}}$ and $\bar{x_{2}}$ are the means of different treatments, and $S_{\bar{x_{1}}-\bar{x_{2}}}$ is the standard error of the mean difference. The significance level was set at p<0.05.

Correlations between microbial species and rumen VFAs were determined using Spearman’s correlation coefficients. Statistical significance was set at p<0.05.

## RESULTS

### Effects of dietary tannic acid supplementation on methane emission

Additionally, the inclusion of tannic acid significantly reduces the daily methane emission of goats, as demonstrated in Table 2. The methane production per DMI in the TAN group is also significantly lower compared to the CON group (p<0.05). However, there is no significant effect on methane emission per ADG. Furthermore, the addition of tannic acid to the diet markedly decreases methane production per unit of neutral detergent fiber intake (NDFI) and acid detergent fiber intake (ADFI) when compared to the CON group (p<0.05). Consequently, the energy of methane is significantly reduced by adding tannic acid to the diet.

### Effects of dietary tannic acid supplementation on apparent nutrient digestibility and growth performance

The addition of tannic acid to the diet of Liuyang black goats has been shown to impact their nutrient digestion and growth performance, as indicated in Table 3. Notably, while the inclusion of tannic acid did not significantly affect the ADG, feed conversion ratio (FCR), or DMI of the goats, some improvements in growth performance were observed (p>0.05). In contrast, the apparent digestibility of nutrients increased with the supplementation of tannic acid. Specifically, there was a significant enhancement in the apparent digestibility of acid detergent fiber (ADF, p<0.05). However, it is important to note that the digestibility of phosphorus significantly decreased with the addition of tannic acid (p<0.05). These findings suggest that while tannic acid may not markedly improve growth metrics, it can enhance the digestibility of certain nutrients, highlighting its potential role in optimizing the nutritional profile of the goats’ diet.

### Effects of dietary tannic acid supplementation on rumen fermentation parameters

The inclusion of tannic acid did not significantly affect the pH and ammonia nitrogen content in the rumen of Liuyang black goats (p>0.05), as shown in Table 4. However, before feeding, the addition of tannic acid led to a significant increase in the concentration of valerate in the rumen fluid of goats (p<0.05), while the ratio of acetate to propionate was significantly decreased (p<0.05). Moreover, 3 h after feeding, the concentrations of both butyrate and valerate were significantly elevated (p<0.05).

### Effects of dietary tannic acid supplementation on diversity of rumen bacterial community

**Figure 1:**
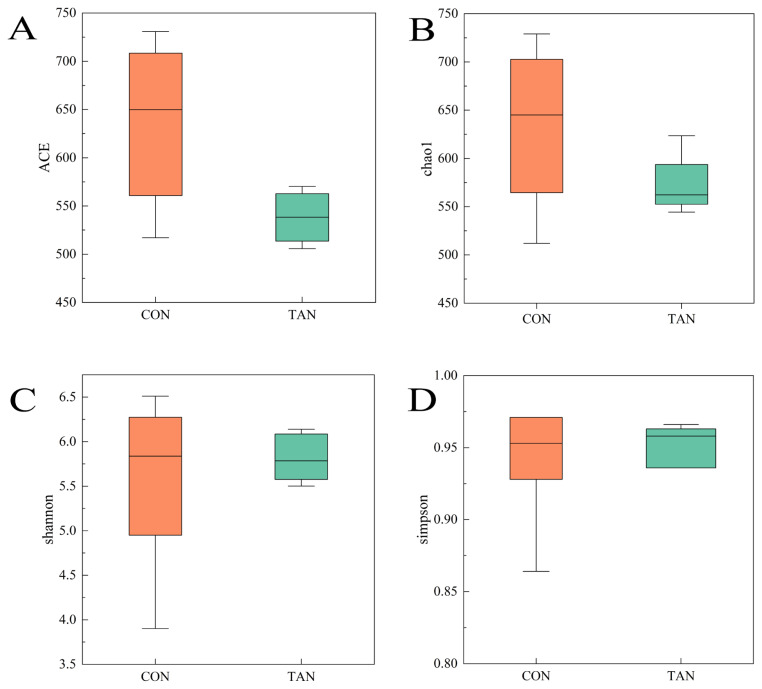
The alpha diversity in the rumen microbial community is based on the observed species. Alpha diversity indexes include (A) the ACE index, (B) the chao1 index, (C) the Shannon index, and (D) the Simpson index. CON, control group; TAN, tannic acid group.

**Figure 2:**
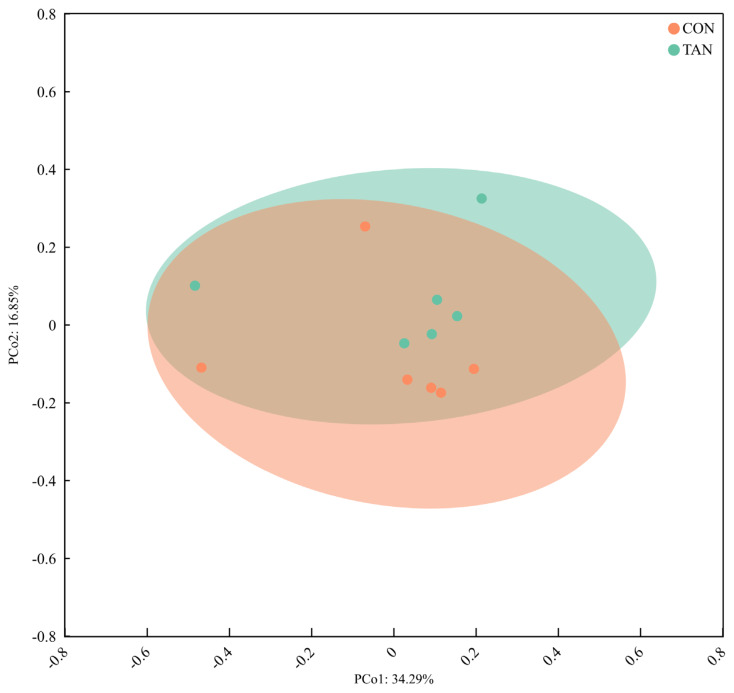
The principal coordinate analysis (PCoA) plots on rumen bacterial community structure across treatments. CON, control group; TAN, tannic acid group.

The Alpha diversity indexes of ACE, Chao1, Shannon, and Simpson for the bacterial microflora in the rumen fluid of Liuyang black goats were all unaffected (p>0.05) by the addition of tannic acid (Figure 1). The results of Beta diversity analysis using weighted UniFrac distance revealed that there was no significant difference in the bacterial community between the CON group and the TAN group (Figure 2).

**Figure 3:**
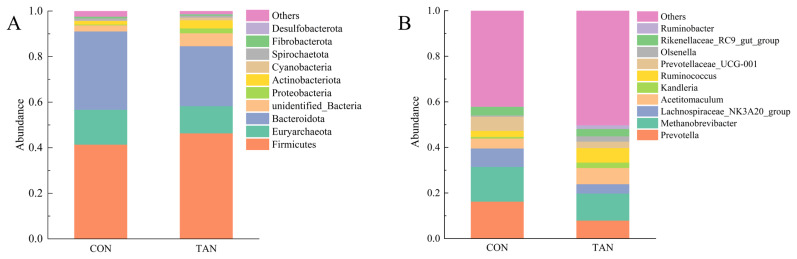
The composition of rumen microbiome at phylum and genus level: the composition of rumen microbiome at phylum level (A), the composition of major rumen genus level (B). CON, control group; TAN, tannic acid group.

Among bacteria, At the phylum level, *Firmicutes* dominated the metagenomic dataset, followed by, *Euryarchaeota*, *Bacteroidetes*, and *unidentified-Bacteria* (Figure 3A). At the genus level, *Methanobrevibacter* was found to be the most abundant, followed by *Prevotella*, *Acetitomaculum*, and *Kandleria* (Figure 3B). *Methanobrevibacter* was more abundant in the CON group than the TAN group. A higher abundance of *Firmicutes* and a lower abundance of *Euryarchaeota* were observed in the TAN group than the CON group (p*<*0.05).

**Figure 4:**
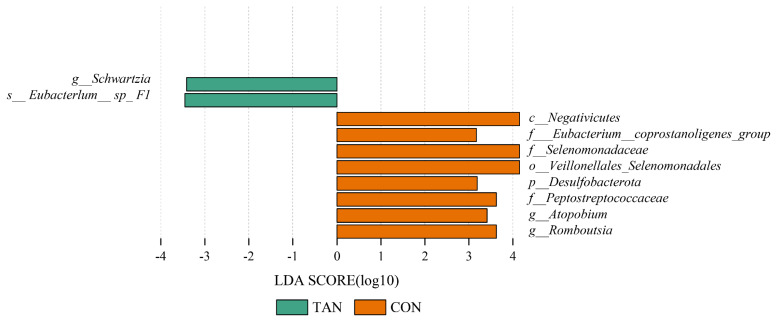
The comparison of the microbial community composition in goat’s rumen using the LEfSe analysis (LDA = 2.0). LDA, linear discriminant analysis; TAN, tannic acid group; CON, control group; LEfSe, linear discriminant analysis effect size.

In addition, a higher abundance of *Schwartzia* was found in the TAN group. In contrast, the CON group exhibited increased levels of *Romboutsia* and *Atopobium*. Additionally, phylum-level analysis revealed a significant presence of *Desulfobacterota* in the CON group (Figure 4). These findings suggest distinct differences in the microbial composition between the treatment groups, highlighting the potential impact of dietary interventions on the gut microbiota of the goats.

**Figure 5:**
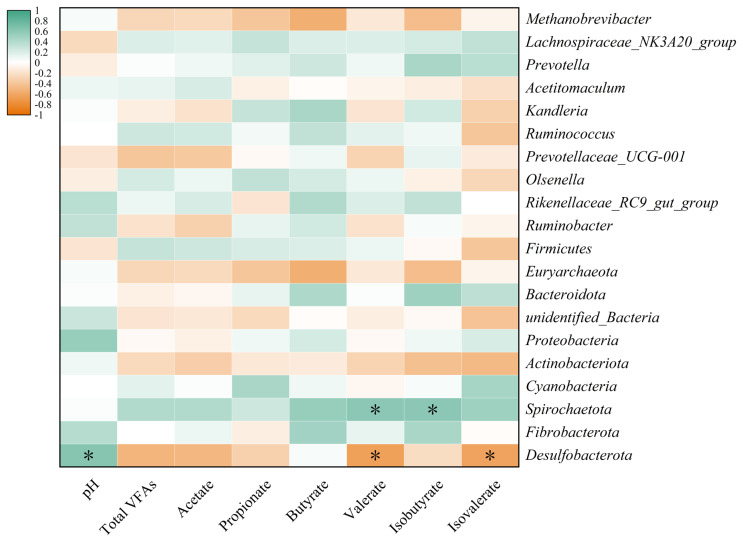
Spearman correlation analysis of genus and phylum microbiota and rumen fermentation parameters. * p<0.05.

Spearman’s correlation analysis was conducted to examine the relationship between the relative abundance of the top 10 genera in the rumen, phylum-level flora, and rumen fermentation characteristics (Figure 5). The results revealed a significant positive correlation (p<0.05) between rumen pH and the presence of *Desulfobacterota* species. In contrast, the ratio of valerate to isovalerate in the ruminal fluid showed a negative correlation (p<0.05) with the relative abundance of *Desulfobacterota*, while exhibiting a positive correlation (p<0.05) with *Spirochaetota*. These findings suggest that specific microbial taxa are associated with variations in rumen fermentation parameters, highlighting the complex interactions within the rumen ecosystem.

## DISCUSSION

Methane production results in significant energy losses during ruminant production. Previous studies have demonstrated that tannins can effectively reduce methane emission from ruminants by altering the fermentation process in the rumen. Specifically, temperate and tropical legumes that are rich in condensed tannins have been shown to significantly lower rumen methane emission [21]. Tiemann et al [22] reported that certain tannin-rich tropical forages exhibit low fiber degradation, which leads to reduced hydrogen production during rumen degradation and a relative increase in rumen pH, ultimately resulting in decreased methane production, which is generally consistent with the results of the present experiment. Furthermore, tannins have an antagonistic effect on proteins, which slows the rate of protein degradation in the rumen, contributing to reduced methane emission. Battelli et al [23] investigated different levels of tannic acid and observed that the 2.0% group produced significantly less methane than the control and lower concentration groups at 12 hours, with the 2.0% and 3.0% groups continuing to show significantly lower methane production after 24 hours. These findings are consistent with our study, indicating that the inclusion of tannic acid in diets significantly reduces gastrointestinal methane emission and minimizes energy losses. Collectively, these findings underscore the potential of various tannin sources and related compounds to effectively mitigate methane emission in ruminant diets, thereby enhancing the sustainability of ruminant production systems.

Tannic acid has traditionally been viewed as antinutritional compounds due to their negative impact on DMI. However, the effects of tannic acid on nutrient intake have been inconsistent across studies. Some experiments have shown that tannic acid levels of 5.5% to 7.5% can inhibit DMI in ruminants, potentially due to reduced feed palatability and the astringency of tannic acid [24]. In contrast, our study found that adding 2% tannic acid to the diet of LiuYang black goats did not significantly affect nutrient intake. This phenomenon may be attributed to the goats’ tolerance for tannic acid, particularly due to the presence of tannin-binding proteins in their saliva [25]. Previous studies have demonstrated varying declines in nutrient digestion among cattle in response to dietary supplementation with condensed or hydrolyzed tannic acid. For instance, Zhang et al [26] reported that supplementing 3% *Acacia mangium* condensed tannic acid reduced the apparent digestibility of DM, NDF, ADF, and CP, while the addition of 3% valonia hydrolyzed tannic acid only decreased CP digestibility in dairy cows. In comparison, research on the impact of tannic acid on nutrient digestibility in goats is limited. Although our study found no significant effect of tannic acid on CP digestion, ADG and FCR of the goats remained unchanged. Interestingly, while our study indicated that supplementing 2% tannic acid increased ADF digestibility in black goats, previous research reported that lower levels of tannic acid (0.3% and 0.6%) resulted in lower ADF digestibility [27]. This discrepancy may be linked to the relatively high proportion of tannic acid used in our study, as well as differences in the types of tannic acid, diets, and animal species involved [28]. Furthermore, the observed increase in ADF digestibility with 2% tannic acid supplementation in our study contrasts with findings from previous research, indicating that the effects of tannic acid are not only dose-dependent but also species-specific. It is possible that the higher concentration of tannic acid utilized in our study may have facilitated an optimal environment for the breakdown of fibrous components, potentially leading to enhanced digestibility.

The ammonia nitrogen is an important indicator of rumen fermentation. If the concentration of ammonia nitrogen in rumen fluid is too high or too low, it will affect the growth and reproduction of rumen microorganisms. The concentration of ammonia nitrogen in the rumen varies between 8.5 and 30 mg per 100 mL. This variation is influenced by several factors, including the degradation rate of dietary protein and non-protein nitrogen (NPN), the capacity of rumen microorganisms to utilize ammonia, and the overall supply of energy and carbon available in the rumen. Understanding these relationships is crucial for optimizing rumen function and improving the efficiency of nutrient utilization in ruminant animals. Previous investigations have demonstrated that the addition of tannic acid at various doses can lower the level of ruminal ammonia nitrogen in beef cattle [4]. This effect is likely due to the ability of tannic acid to bind with feed proteins, protecting them from microbial degradation in the rumen [29]. In the current study, a diet supplemented with 2% tannic acid also showed a downward trend in ammonia nitrogen concentration, although the changes were not statistically significant. This suggests that tannic acid may help optimize ammonia nitrogen levels by reducing protein degradation, thereby improving the efficiency of nitrogen utilization.

In addition to ammonia nitrogen, VFAs play a crucial role as indicators of rumen fermentation in sheep. They reflect the environmental conditions within the rumen, the extent of fermentation, and the type of feed consumed [30]. In the current study, the addition of tannic acid significantly increased total short-chain fatty acids, particularly valerate concentrations at both 0 h and 3 h post-feeding. This increase in VFAs concentrations, despite no significant changes in DMI and nutrient digestibility, may be attributed to tannic acid’s ability to slow down the rate of digesta evacuation from the rumen. This slower evacuation rate can reduce the clearance of VFAs, leading to higher ruminal VFAs concentrations [29]. Furthermore, the increased concentration of total volatile fatty acids (TVFAs), particularly valerate, suggests that tannic acid may influence the fermentation profile within the rumen. Valerate is known to be associated with the fermentation of certain fibrous feeds, indicating that the presence of tannic acid could enhance the breakdown of fibrous components, thereby promoting the production of specific TVFAs that serve as energy sources for the host animal [31]. Moreover, the accumulation of VFAs in the rumen can have several physiological implications for the host. Elevated levels of TVFAs can enhance ruminal pH stability, which is essential for maintaining a healthy rumen environment and supporting optimal microbial activity. This stability in pH not only fosters a conducive environment for beneficial microorganisms but also helps prevent the proliferation of harmful pathogens, ultimately promoting overall digestive health and efficiency in ruminants [32]. Interestingly, while our findings align with previous studies indicating that tannic acid can influence VFAs production, the specific effects observed may vary based on the dosage and type of tannic acid used, as well as the overall dietary composition.

The pH value is a crucial indicator of rumen fermentation levels. It can be influenced by various factors, including salivary secretion, diet composition, and the composition of rumen microbiota. Previous studies have demonstrated that under *in vitro* culture conditions, higher concentrations of tannic acid lead to a slower decrease in the pH of the culture medium. One possible explanation for this phenomenon is that tannins inhibit the fermentative activity of rumen microorganisms, which in turn reduces the overall rate of fermentation. This inhibition contributes to stabilizing the pH of the rumen, thereby minimizing the accumulation of substances that could be detrimental to microbial activity [33]. Additionally, a decrease in CP deamination may lead to lower ammonia release, which further contributes to a reduction in ruminal pH [34]. In this experiment, the pH values were maintained within the normal range, and no significant difference was observed between the fermentation broth pH values of the TAN group and the CON group. This indicates that the addition of tannic acid did not adversely affect the ruminal pH, supporting the notion that they help stabilize the fermentation environment.

In this study, the addition of tannic acid did not significantly affect of the alpha - diversity indices of rumen bacteria. This finding suggests that tannic acid does not substantially alter the species’ richness and homogeneity of rumen bacterial communities. Conversely, the beta - diversity analysis, assessed through weighted UniFrac distance, also revealed no significant differences in bacterial community composition between the CON group and the TAN group. This further indicates that tannic acid has a limited impact on the overall structure of the rumen bacterial community. These results imply that while tannic acid may not broadly affect the diversity or overall composition of the rumen microbial community, it can selectively modulate the growth and metabolic activity of specific microbial populations. At the genus level, *Methanobrevibacter* was the microorganism with the highest relative abundance in the rumen, which is consistent with the results of previous studies [35]. *Methanobrevibacter* is a major contributor to rumen methane emission in the rumen, mainly through methane synthesis using hydrogen and formic acid [36]. In this study, we found that the relative abundance of *Methanobrevibacter* was higher in the CON group than in the TAN group, which may be related to the inhibitory effect of tannic acid on hydrogen production. Tannins are able to bind to proteins to form complexes, which reduces the degradability of proteins and thus reduces hydrogen production from microbial metabolism [37]. Since *Methanobrevibacter* depends on hydrogen for growth and metabolism, a decrease in hydrogen may have resulted in inhibition of its growth and thus a decrease in its relative abundance. In addition, the higher relative abundance of *Schwartzia* in the TAN group may be related to the addition of tannic acid. *Schwartzia* is a microorganism capable of utilizing tannins for growth, and its increase in the TAN group may reflect the fact that tannins provide an additional growth substrate for this microorganism. At the phylum level, *Firmicutes* is the dominant phylum in the rumen microbial community, which is in line with the results of most studies of rumen microbes in ruminants [6]. *Firmicutes* are mainly involved in the degradation of cellulose and starch in the rumen, which provide energy and nutrients to the host animal [38]. In this study, we found that the relative abundance of Firmicutes in the TAN group was higher than that in the CON group (p<0.05), which may be related to the promoting effect of tannic acid on cellulolytic bacteria. Tannic acid can bind to cellulose and change the structure of cellulose, making it easier to be decomposed by microorganisms. Thus, the addition of tannic acid may have promoted the growth of cellulolytic bacteria in Firmicutes, thereby increasing their overall relative abundance.

In addition, this study also found that the relative abundance of *Desulfobacterota* was significantly higher in the CON group than in the TAN group. *Desulfobacterota* are a group of microorganisms capable of sulfate reduction, and their presence in the rumen may be related to the production of sulfate during rumen fermentation. By reducing sulfate to hydrogen sulfide, sulfate-reducing bacteria are able to consume hydrogen in the rumen, thereby reducing methane production [39]. Therefore, the high abundance of *Desulfobacterota* in the CON group may reflect its effective utilization of hydrogen during rumen fermentation, which in turn inhibits methane production. However, the low relative abundance of *Desulfobacterota* in the TAN group may be related to the interference of tannins with the sulfate reduction process. Tannic acid may reduce their relative abundance by competing with sulfate-reducing bacteria for substrates or inhibiting their metabolic activity. The results of the Spearman correlation analysis further revealed the complex relationship between the structure of the rumen microbial community and rumen fermentation characteristics. The study found a significant positive correlation (p<0.05) between rumen pH and the relative abundance of *Desulfobacterota*, indicating that *Desulfobacterota* may thrive better in environments with higher pH [40]. Elevated pH levels may enhance the activity of sulfate-reducing bacteria, thereby promoting their growth and metabolism. Conversely, valeric acid and isovaleric acid in the rumen fluid showed a significant negative correlation (p<0.05) with the relative abundance of *Desulfobacterota*, while exhibiting a significant positive correlation (p<0.05) with the relative abundance of *Spirochaetota*. This may reflect the varying contributions of different microorganisms to the metabolism of VFAs during rumen fermentation [40]. The valeric acid and isovaleric acid are typically associated with the degradation levels of cellulose and starch in the rumen. A lower ratio may indicate relatively less cellulose degradation and more starch degradation in the rumen, which could be unfavorable for the growth of *Desulfobacterota* but beneficial for *Spirochaetota*.

This present study demonstrated that while the addition of tannic acid had a limited effect on the alpha-diversity and overall structure of rumen bacteria, it significantly influenced the relative abundance of certain microorganisms. Additionally, there is a complex interplay between the structure of the rumen microbial community and fermentation characteristics, with changes in the relative abundance of specific taxa closely linked to variations in rumen fermentation parameters. These findings improve our understanding of how tannic acid affects rumen microbial communities and fermentation in ruminants, providing a theoretical foundation for developing tannin-based feed additives to enhance ruminant performance and reduce methane emission.

## CONCLUSION

Adding tannic acid to the diet of Liuyang black goats resulted in a decrease in methane emission in rumen. Additionally, it led to a decrease in the ratio of acetic acid to propionic acid in the rumen fluid, a reduction in the amount of valeric acid, and a slight increase in the digestibility of ADF was observed. The findings suggest that tannic acid could be a potential feed additive for mitigating methane emission and improving feed efficiency in ruminants. Further research is needed to explore the long-term effects of tannic acid on goat performance and health.

## Figures and Tables

**Table 1:** Composition and nutrition level of the experiment diets (DM basis, %)

Items	Treatments1)	

	CON	TAN
Ingredients		
Corn straw	17.76	17.76
Soy husk	10.34	10.34
Sunflower hull	2.00	2.00
Corn	48.60	48.60
Soybean meal	10.55	10.55
Sesame cake	3.00	3.00
Molasses	5.00	3.00
Limestone	0.25	0.25
Expanded urea	0.80	0.80
Salt	0.70	0.70
Vitamin and mineral mix2)	1.00	1.00
Tannic acid	0.00	2.00
Total	100.00	100.00
Nutrient contents3)		
DE (MJ/kg)	12.31	12.10
CP (%)	14.92	14.18
NDF (%)	49.22	49.10
ADF (%)	21.95	21.77
Ca (%)	0.31	0.31
P (%)	0.21	0.21

1) Control group (CON); tannic acid group (TAN).

2) Contained per kilogram of supplement: Fe (mg) = 25, Mn (mg) = 40, Zn (mg) = 40, Cu (mg) = 8, I (mg) = 0.3, Se (mg) = 0.3, Co (mg) = 0.1, Vit A (IU) = 940, Vit D (IU) = 111, Vit E (IU) = 20.

3) Digestible energy (DE), the digestive energy in the nutritional level was calculated, and the rest were measured; crude protein (CP), neutral detergent fiber (NDF), acid detergent fiber (ADF), calcium (Ca), phosphorus (P).

DM, dry matter.

**Table 2:** Effects of tannic acid on methane emission in goats

Items1)	Treatment2)		SEM	p-value

	CON	TAN		
CH_4_ (g/d)	10.80	4.99	1.35	0.040
CH_4_ (g/kg DMI)	16.78	7.16	1.75	0.004
CH_4_ (g/kg ADG)	48.89	31.96	7.16	0.260
CH_4_ (g/kg NDFI)	32.08	13.19	3.41	0.003
CH_4_ (g/kg ADFI)	69.01	29.69	7.17	0.004
CH_4_ (MJ/d)	0.60	0.28	0.07	0.040

1) CH_4_, daily methane emission; CH_4_/DMI, methane emission from dry matter intake; CH_4_/ADG, methane emission from average daily gain; CH_4_/NDFI, methane emission per neutral detergent fiber intake; CH_4_/ADFI, methane emission per acid detergent fiber intake; CH_4_-E, methane energy.

2) Control group (CON); tannic acid group (TAN).

SEM, standard error of the mean.

**Table 3:** Effects of tannic acid supplementation on apparent digestibility of nutrients in goats

Items	Treatment1)		SEM	p-value

	CON	TAN		
Growth performance				
DMI (g/d)	674.50	713.56	63.82	0.775
ADG (g/d)	192.10	206.43	22.14	0.763
F/G	4.01	4.72	0.66	0.616
Apparent nutrient digestibility (%)				
DM	65.83	73.00	0.03	0.215
NDF	28.67	45.00	0.05	0.072
ADF	26.17	34.67	0.02	0.010
CP	57.33	67.33	0.03	0.066
Ca	33.83	36.67	0.03	0.553
P	42.00	38.67	0.04	0.001

1) Control group (CON); tannic acid group (TAN).

SEM, standard error of the mean; DMI, dry matter intake; ADG, average daily gain; F/G, feed to gain ratio; DM, dry matter; NDF, neutral detergent fiber; ADF, acid detergent fiber; CP, crude protein.

**Table 4:** Effect of tannic acid on ruminal pH, ammonia nitrogen, volatile fatty acids in goats

Items	Treatment1)		SEM	p-value

	CON	TAN		
Before feeding				
pH	6.56	6.48	0.06	0.534
Ammonia nitrogen (mg/100 mL)	10.81	10.71	0.98	0.962
TVFAs (mM)	58.36	68.63	5.18	0.346
VFA proportion (mol/100 mol)				
Acetate	60.27	58.18	1.04	0.340
Propionate	19.65	19.49	1.32	0.957
Butyrate	15.69	17.79	0.70	0.137
Valerate	0.76	1.39	0.14	0.010
Isobutyrate	1.37	1.04	1.31	0.215
Isovalerate	2.27	2.12	0.25	0.767
Acetate/propionate	3.18	1.82	0.33	0.032
3 h after feeding				
pH	5.94	5.82	0.08	0.635
Ammonia nitrogen (mg/100 mL)	13.46	9.93	1.23	0.161
TVFAs (mM)	111.60	111.89	5.27	0.948
VFA proportion (mol/100 mol)				
Acetate	53.01	53.95	1.36	0.748
Propionate	32.35	26.47	1.64	0.070
Butyrate	13.12	17.16	0.81	0.006
Valerate	0.70	1.61	0.16	0.001
Isobutyrate	0.30	0.31	0.04	0.805
Isovalerate	0.44	0.50	0.06	0.679
Acetate/propionate	1.75	2.09	0.17	0.338

1) Control group (CON); tannic acid group (TAN).

SEM, standard error of means for treatments; TVFAs, total volatile fatty acids; VFA, volatile fatty acid.

## Data Availability

Upon reasonable request, the datasets of this study can be available from the corresponding author.
